# AER-Net: Attention-Enhanced Residual Refinement Network for Nuclei Segmentation and Classification in Histology Images

**DOI:** 10.3390/s24227208

**Published:** 2024-11-11

**Authors:** Ruifen Cao, Qingbin Meng, Dayu Tan, Pijing Wei, Yun Ding, Chunhou Zheng

**Affiliations:** 1 The Information Materials and Intelligent Sensing Laboratory of Anhui Province, School of Computer Science and Technology, Anhui University, Hefei 230601, China; 2 Institutes of Physical Science and Information Technology, Anhui University, Hefei 230601, China; 3 School of Artificial Intelligence, Anhui University, Hefei 230601, China

**Keywords:** histology images, deep learning, nuclei segmentation, nuclei classification

## Abstract

The acurate segmentation and classification of nuclei in histological images are crucial for the diagnosis and treatment of colorectal cancer. However, the aggregation of nuclei and intra-class variability in histology images present significant challenges for nuclei segmentation and classification. In addition, the imbalance of various nuclei classes exacerbates the difficulty of nuclei classification and segmentation using deep learning models. To address these challenges, we present a novel attention-enhanced residual refinement network (AER-Net), which consists of one encoder and three decoder branches that have same network structure. In addition to the nuclei instance segmentation branch and nuclei classification branch, one branch is used to predict the vertical and horizontal distance from each pixel to its nuclear center, which is combined with output by the segmentation branch to improve the final segmentation results. The AER-Net utilizes an attention-enhanced encoder module to focus on more valuable features. To further refine predictions and achieve more accurate results, an attention-enhancing residual refinement module is employed at the end of each encoder branch. Moreover, the coarse predictions and refined predictions are combined by using a loss function that employs cross-entropy loss and generalized dice loss to efficiently tackle the challenge of class imbalance among nuclei in histology images. Compared with other state-of-the-art methods on two colorectal cancer datasets and a pan-cancer dataset, AER-Net demonstrates outstanding performance, validating its effectiveness in nuclear segmentation and classification.

## 1. Introduction

Colorectal cancer is recognized as one of the most prevalent malignant tumors, with various factors, including genetic and environmental influences, potentially leading to its development [1]. The pathological analysis and diagnosis of colorectal cancer based on hematoxylin and eosin (H&E)-stained histology images have become the gold standard in clinical practice. However, manual pathological analysis not only depends on expert experience but also is time consuming, labor-intensive, and inefficient. In order to overcome the difficulties of visual evaluation of tissue sections, there is increasing interest in digital pathology (DP), where scanning devices are used to obtain digital whole slide images (WSI) from glass tissue sections. This allows for the effective processing, analysis, and management of tissue specimens [2]. There are numerous nuclei of different types in each whole slide image. The precise segmentation and classification of nuclei that are interested play a vital role in improving the quantification of WSIs, offering critical information for tasks like cancer diagnosis and predicting clinical outcomes [3,4]. The segmentation and classification of nuclei is mainly to distinguish different nuclei on the basis of various morphological features, such as the shape, contour, size, color and texture features of nuclei. This study focuses on the segmentation and classification of miscellaneous, inflammatory, epithelial, and spindle-shaped nuclei in pathological images of colorectal cancer.

**Figure 1:**
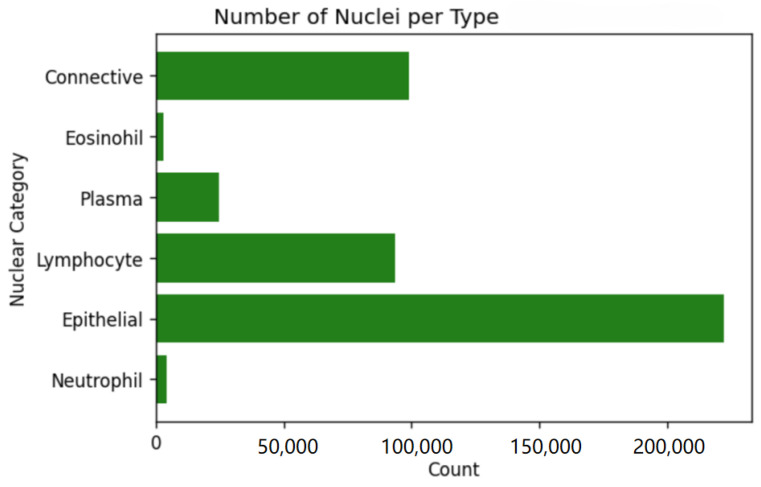
The number of nuclei per type in the CoNIC2022 dataset.

With the advancement of deep learning technology, methods utilizing deep learning have exhibited outstanding performance in both the segmentation and classification of nuclei. Notably, Graham et al. [5] proposed a deep learning framework based on vertical and horizontal distance maps to simultaneously segment and classify instances in histopathological images. However, there are numerous nuclear clusters in histopathological images, and it is difficult to accurately predict the nuclear boundaries and types in these regions. In addition, the number of nuclei varies significantly across different types. The problem of class imbalance in the task of segmenting and classifying nuclei in pathology images refers to the significant difference in the number of different types of nuclei. For example, in the CoNIC Data we used, the number of nuclei corresponding to the six types is shown in Figure 1 [6], indicating a serious imbalance between the samples. This class imbalance can adversely affect the algorithm’s performance, especially when processing classes with a small number of instances. In this paper, we propose a novel attention-enhanced residual refinement network (AER-Net), which consists of one encoder and three decoder branches that have the same network structure. The AER-Net utilizes an attention-enhanced encoder module to focus on more valuable features. To further refine predictions and achieve more accurate results, an attention-enhancing residual refinement module is employed at the end of each encoder branch. Moreover, the coarse predictions and refined predictions are combined by the loss function that employs cross-entropy loss and generalized dice loss to efficiently tackle the challenge of class imbalance among nuclei in histology images. We conducted experiments using three datasets, and our method demonstrated superior performance compared with other state-of-the-art approaches. The contributions of this study are summarized below:

1. To enhance the ability of the AER-Net encoder extracting nuclei features in pathology images, a spatial and channel attention enhancement encoder module is utilized to improve nuclei segmentation and classification performance.
2. To address the challenge of accurately predicting nuclear boundaries, we integrate an attention-enhanced residual refinement module into our nuclear segmentation and classification approach. This enhancement enables the model to produce more precise predictions, and experimental results confirm the effectiveness of this attention-enhanced residual refinement module.
3. A loss function that not only combines cross-entropy loss and generalized dice loss but also integrates coarse predictions and refined predictions is utilized to mitigate the class imbalance of nuclei and improve the overall results.

## 2. Related Work

### 2.1. Nuclei Segmentation Methods

For nuclei segmentation, there exist both traditional methods and deep learning-based approaches. Numerous methods based on traditional image processing techniques have been proposed for nuclear segmentation. Yang et al. [7] proposed a marker-controlled watershed based on mathematical morphology. It first utilizes a thresholding method to obtain markers and energy landscapes and then inputs them into the watershed algorithm to obtain segmentation results. Ali et al. [8] proposed a synergistic boundary and region-based active contouring model that combines an active contours approach with nuclear shape modeling. Consequently, it obtains the final segmentation result by a level-set method. Active contours is a classical image segmentation method. It is based on the mathematical idea called “energy minimization”, which iteratively adjusts the contour lines to better fit the boundary of the segmentation target [9]. The level-set method is a mathematical method that can be used to track the evolution of curves and surfaces and is widely used in the field of image segmentation.

However, the performance of these methods is highly dependent on the choice of parameters, and they are not highly generalizable. Deep learning models can automatically learn image features, and many excellent deep learning models have emerged in computer vision tasks. In recent years, deep learning models have made tremendous progress in the field of medical image processing. Ronneberger et al. [10] proposed U-Net, which has been successfully applied to various medical segmentation tasks. Inspired by UNet, Micro-Net [11] was proposed by Raza et al., which is an architecture for segmenting nuclei and glands in multi-resolution histology images. Chen et al. [12] proposed a deep contour-aware network (DCAN) with two independent branches for predicting instances and contours simultaneously. Zhou et al. [13] proposed CIA-Net, which utilizes a multi-level information aggregation module between two task-specific decoders. These decoders separately output nuclei and contour predictions, enabling the full utilization of the spatial and textural dependencies between nuclei and contours. Naylor et al. [14] used distance map regression for segmentation to split adjacent nuclei. He et al. proposed Mask-RCNN [15] for instance segmentation. It is a two-stage model that initially predicts the candidate regions and then inputs the proposed regions into the segmentation module to obtain the segmentation results. Currently, researchers have already applied Mask-RCNN, an instance segmentation model, to the task of nuclei segmentation [16].

### 2.2. Nuclei Classification Methods

For nuclear classification methods, many previous nuclear classification methods typically involve two models, the first for nuclear detection and the second for nuclear classification. Yuan et al. [17] utilized the AdaBoost classifier to classify each nucleus based on intensity, morphology, and texture as feature information. Wang et al. [18] proposed a mitotic detection cascade approach that combines CNN-extracted features and hand-crafted features to classify mitotic and anaphase nuclei. Sirinukunwattana et al. [19] proposed a Spatially Constrained Convolutional Neural Network to detect nuclei and a Neighborhood Ensemble Predictor to further predict the class of nuclei. Rahaman et al. [20] proposed a DL-based hybrid depth feature fusion (HDFF) technique for the accurate classification of cervical cytopathic cells. Lately, there have been several methods to segment and classify nuclei simultaneously.

### 2.3. Simultaneous Nuclei Segmentation and Classification Methods

In recent years, methods for the simultaneous nuclear segmentation and classification of pathology images have emerged in this field. Graham et al. [5] proposed a deep learning method that allows for the simultaneous segmentation and classification of nuclei. This method utilizes horizontal and vertical distance maps of predicted nuclear pixels to the center of mass of that nucleus. It obtains the results of nucleus instance segmentation via a watershed algorithm and predicts the class of nucleus by a separate classification branch. Xiao et al. [21] proposed a scale and regional enhanced decoder network for nuclei classification in histology images, which improves classification accuracy by enhancing nuclear region information. Powerful representations in encoders are essential for the segmentation and classification of nuclei. However, the methods described above do not specifically design a suitable encoder for these tasks. The above methods do not use the prediction refinement module to improve the performance of the methods in nuclear segmentation and classification tasks. In the task of nuclei segmentation and classification of pathology images, nuclei class imbalance is the situation where some types of nuclei are much more numerous than others. This is very common in the real pathology datasets and can pose a challenge for the model to predict the nuclei of the lesser numbered types. The class imbalance problem in the nuclear classification task needs to be further addressed.

## 3. Methodology

**Figure 2:**
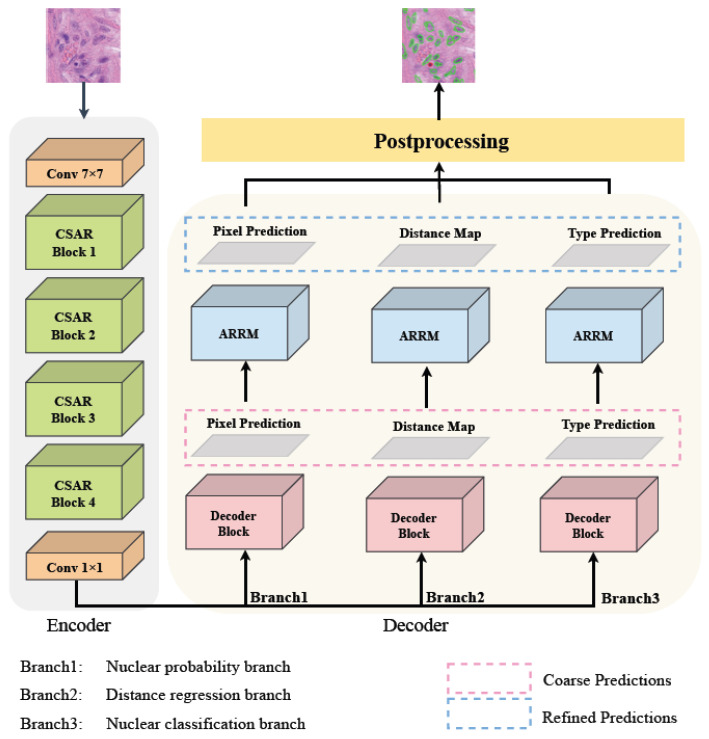
Illustration of overall architecture.

The AER-Net employs a classical encoder–decoder architecture. The model comprises three decoder branches: a nuclear probability branch, a distance map regression branch, and a nuclear classification branch. All of these branches share an attention-augmented encoder, as illustrated in Figure 2. AER-Net initially predicts the distance map and probability map to segment the instances of nuclei, and it subsequently integrates the predictions from the classification prediction branch to predict the types of nuclei. AER-Net utilizes spatial and channel attention modules within the encoder to enhance the representational power of the extracted features. An attention-enhanced residual refinement module is added to the end of each branch of the decoder to achieve accurate nuclear boundaries and class predictions.

### 3.1. Encoder

**Figure 3:**
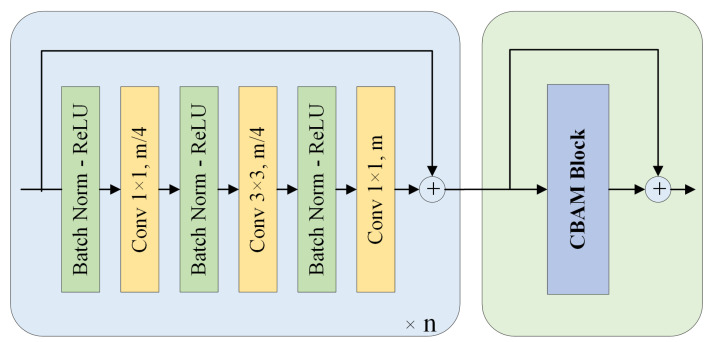
Illustration of CSAR block.

The model employs an encoder module enhanced with channel and spatial attention, as illustrated in Figure 3. Inspired by ResNet [22] and CBAM [23], we propose a channel and spatial attention-enhanced residual (CSAR) encoder block, where *n* represents the number of residual units, and the corresponding *n* values for CSAR blocks 1 to 4 are 3, 4, 6, and 3, respectively. Specifically, we improve the ResNet-50 network by removing the initial max pooling module and setting the stride of the first convolutional layer to 1, aiming to obtain as many detailed features of the nuclei as possible. In addition, we add channel and spatial attention modules at the end of each stage of ResNet-50. The addition of the channel and spatial attention modules enhances the encoder’s representation, which is important for predicting nuclear contours and classes. The role of the channel attention module is to allow the model to dynamically adjust the weights of each channel based on the importance of the input features. The spatial attention module computes attention on the spatial dimension of the feature map to focus on regions of the feature map that are more important to the task. The attention mechanism allows the model to pay more attention to the feature information in the nuclear dense region and reduce the influence of other interfering information.

### 3.2. Decoder

**Figure 4:**
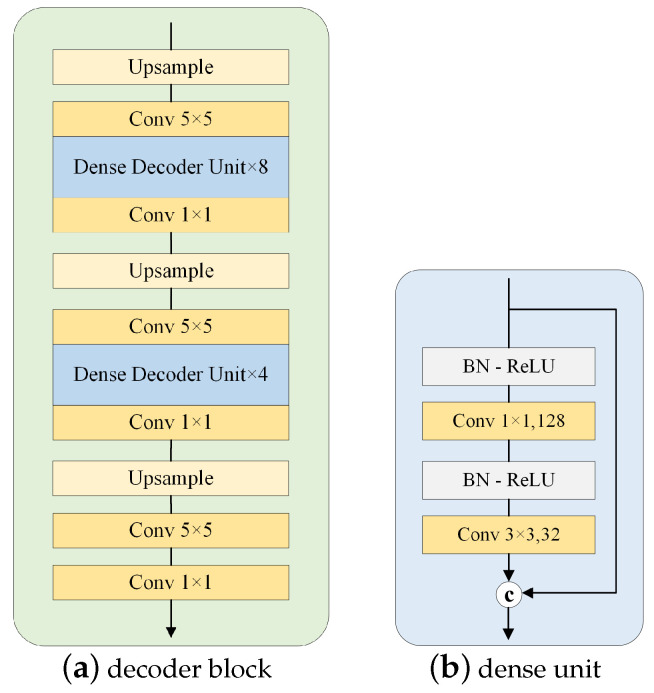
The structure of the decoder block.

There are three decoder branches in our model: the nuclear probability branch, the distance map regression branch, and the nuclear classification branch, as shown in Figure 1. Inspired by HoverNet [5], we employ the same structure for the three decoder branches, which include upsampling operations and dense units [24]. The decoder block is illustrated in Figure 4, with dense units employed after the first and second upsampling, utilizing 8 and 4 dense units, respectively. We use a skip connection to merge the features of the encoder. Specifically, we add the feature maps of each CSAR module in the encoder to the corresponding feature maps in the decoder. This approach optimizes the utilization of the detailed information of the low-level features and enables the decoder to better recover details of nuclei. It is worth noting that the feature maps provided by the CSAR module are augmented by the channel and spatial attention modules, and the CSAR module suppresses irrelevant information and enhances information on features important for the task of nuclear segmentation and classification. After each decoder block, we add an attention-enhanced residual refinement block to obtain refined prediction results.

### 3.3. The Attention-Enhanced Residual Refinement Module

**Figure 5:**
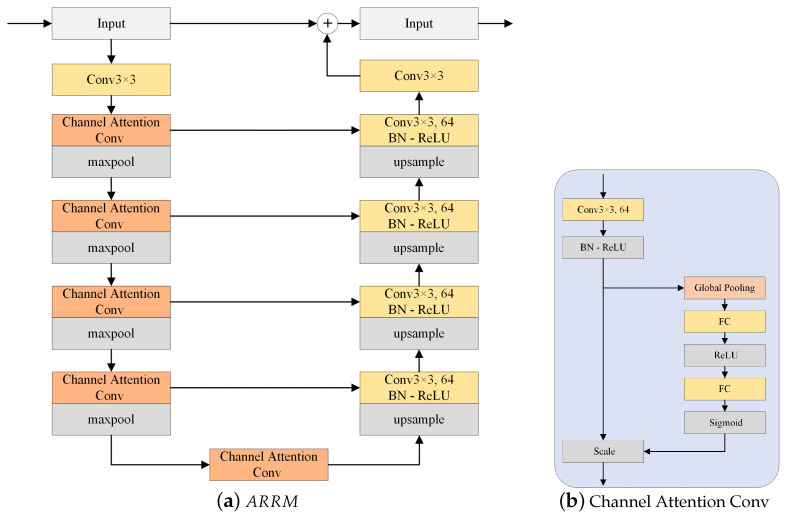
The structure of the attention-enhancing residual refinement module.

The representation of different types of nuclei in pathological tissue images is variable. Tumor nuclei frequently appear as clusters, making the boundaries of these nuclei difficult to predict accurately. Inspired by the works of Qin et al. [25] and Hu et al. [26], we propose an attention-enhanced residual refinement module (ARRM) to address the above problem, as illustrated in Figure 5a. The ARRM is designed with an encoder–decoder architecture, where the encoder and decoder modules have different designs. The encoder is composed of a convolutional layer and four stages, and each stage includes a channel attention convolution module and a max pooling layer. As shown in Figure 5b, the channel attention convolution module is composed of a 3×3 convolution layer followed by a batch normalization and ReLU activation function and a channel attention mechanism. Correspondingly, the decoder consists of four stages and a single 3×3 convolutional layer. Each stage in the decoder consists of a 3×3 convolutional layer, which is followed by batch normalization and a ReLU activation function. In the attention-enhanced residual refinement module, a channel attention convolution module is used as a bridging module between the encoder and decoder. The residual refinement module allows further adjustments to the coarse predictions to obtain more accurate refined predictions. The initial segmentation results may have rough boundaries and missing details. The refinement module can optimize the boundary of the segmented region by finer feature extraction.

### 3.4. Loss Function

The total loss function of the proposed network is the sum of the auxiliary loss $L_{1}$ multiplied by the weight α and the final loss $L_{2}$.

(1) $$\begin{array}{c} L=\alpha L_{1}+L_{2} \end{array}$$

where $L_{1}$ is the loss calculated by coarse predictions and $L_{2}$ is the loss calculated by the refined predictions using ARRM. The model has three decoder branches, and each branch uses a different loss function.

(2) $$\begin{array}{c} L_{i}=L_{i,a}+L_{i,b}+L_{i,c},i\in \left\{1,2\right\} \end{array}$$

where $L_{i,a}$ is the loss function of the nuclear probability branch, $L_{i,b}$ is the loss function of the distance regression branch, and $L_{i,c}$ is the loss function of the nuclear classification branch.

#### 3.4.1. Distance Regression Branch

The loss function $L_{b}$ of the distance map regression branch is as follows:

(3) $$\begin{array}{c} L_{b}=\beta_{1}L_{mse}+\beta_{2}L_{msge} \end{array}$$

(4) $$\begin{array}{c} L_{mse}=\frac{1}{N}\sum_{i=1}^{N}{\left(X_{i}-{\hat{X}}_{i}\right)}^{2} \end{array}$$

(5) $$\begin{array}{c} L_{msge}=\frac{1}{m}\sum_{i\in M}{\left(\nabla_{x}\left(X_{i,x}\right)-\nabla_{x}\left({\hat{X}}_{i,x}\right)\right)}^{2}\\ +\frac{1}{m}\sum_{i\in M}{\left(\nabla_{y}\left(X_{i,y}\right)-\nabla_{y}\left({\hat{X}}_{i,y}\right)\right)}^{2} \end{array}$$

where $L_{mse}$ is the mean squared error loss, $L_{msge}$ is the gradient mean squared error loss, and $\beta_{1}$ and $\beta_{2}$ are the corresponding weights of $L_{mse}$ and $L_{msge}$. $\beta_{1}$ and $\beta_{2}$ are 1 by default. $\hat{X_{i}}$ denotes the ground truth of the horizontal and vertical distances of the nuclear pixels to their corresponding centers of mass, and $X_{i}$ is the horizontal and vertical distance map predicted by the network. $\nabla_{x}$ and $\nabla_{y}$ represent the horizontal and vertical gradients, respectively. *M* represents the set that contains all nuclear pixels, and *m* represents the number of nuclear pixels in the image.

#### 3.4.2. Nuclear Probability Branch

The loss function $L_{a}$ of the nuclear probability branch is as follows:

(6) $$\begin{array}{c} L_{a}=\beta_{3}L_{ce}+\beta_{4}L_{dice} \end{array}$$

(7) $$\begin{array}{c} L_{dice}=1-\frac{2*\sum_{i=1}^{N}\left(X_{i}*{\hat{X}}_{i}\right)+\varepsilon }{\sum_{i=1}^{N}X_{i}+\sum_{i=1}^{N}{\hat{X}}_{i}+\varepsilon } \end{array}$$

(8) $$\begin{array}{c} L_{ce}=-\frac{1}{N}*\sum_{i=1}^{N}\sum_{m=1}^{M}X_{i,m}*log{\hat{X}}_{i,m} \end{array}$$

where $\hat{X}$ is defined as the ground truth, *X* is the predicted outcome, *i* is the pixel of *X* and $\hat{X}$, and ε is a constant set to 0.001. $L_{ce}$ and $L_{dice}$ denote cross-entropy loss and dice loss, respectively. $\beta_{3}$ is the weight of $L_{ce}$ and $\beta_{4}$ is the weight of $L_{dice}$. $\beta_{3}$ and $\beta_{4}$ are 1 by default.

#### 3.4.3. Nuclear Classification Branch

In the classification branch, we utilize a loss function that combines cross-entropy loss and generalized dice loss. The class imbalance in nuclear segmentation and classification datasets tends to make it challenging to detect nuclei in classes with a small number of instances. Although Graham et al. [5] use the sum of cross-entropy and dice loss as a loss function in the nuclei classification branch, addressing class imbalance remains a challenge. We utilize the sum of the cross-entropy loss and generalized dice loss in the classification branch to address the problem of class imbalance. The loss function $L_{c}$ of the nuclear classification branch is as follows:

(9) $$\begin{array}{c} L_{c}=\beta_{5}L_{ce}+\beta_{6}GDL \end{array}$$

(10) $$\begin{array}{c} GDL=1-2\frac{\sum_{l=1}^{K}w_{l}\sum_{n}r_{ln}p_{ln}}{\sum_{l=1}^{K}w_{l}\sum_{n}\left(r_{ln}+p_{ln}\right)} \end{array}$$

(11) $$\begin{array}{c} w_{l}=\frac{1}{{\left(\sum_{n=1}^{N}r_{ln}\right)}^{2}} \end{array}$$

where $L_{ce}$ is the cross-entropy loss function, GDL is the generalized dice loss [27], and $\beta_{5}$ and $\beta_{6}$ are the corresponding weights, respectively. $\beta_{5}$ and $\beta_{6}$ are 1 by default. *K* is the number of classes and $w_{l}$ is the weight of the class *l* as shown in Equation (11). $r_{ln}$ represents the true label value, and $p_{ln}$ represents the predicted probability value. Since GDL assigns higher weights to classes with fewer nuclei, it makes the model pay more attention to a few classes, thus effectively addressing the problem of class imbalance.

### 3.5. Post-Processing

Inspired by HoverNet [5], we employ the following post-processing method. In the horizontal and vertical distance maps, the pixel differences between different cell nuclei are significant. Therefore, we compute the gradients of the predicted horizontal and vertical distance maps, and the generated gradient maps can highlight the regions where there are significant differences between neighboring pixels.

(12) $$\begin{array}{c} S_{m}=max\left(H_{x}\left(p_{x}\right),H_{y}\left(p_{y}\right)\right) \end{array}$$

where $p_{x}$ and $p_{y}$ are the results of the horizontal and vertical distance maps output by the distance regression branch, and $H_{x}$ and $H_{y}$ are the horizontal and vertical components of the Sobel operator. $S_{m}$ highlights the regions where there are significant differences between neighboring pixels in the horizontal and vertical ranges. The markers *M* and energy landscape *E* are computed as follows:

(13) $$\begin{array}{c} M=\sigma (\tau \left(q,h\right)-\tau \left(S_{m},k\right)) \end{array}$$

(14) $$\begin{array}{c} E=[1-\tau \left(S_{m},k\right)*\tau \left(q,h\right)] \end{array}$$

where *q* is the prediction result of the nuclear probability branch. τ(a,b) is the threshold function operating on the matrix *a*, which outputs 1 when the element is greater than *b* and 0 otherwise. *h* and *k* are set according to experience. σ changes all negative values to 0. After obtaining *M* and *E*, nuclear instance segmentation results could be obtained using the marker-controlled watershed algorithm. Finally, the instance segmentation results are combined with the output of the nuclei classification branch to perform class majority voting on the pixels within each instance of cell nuclei to obtain the final nuclei segmentation and classification prediction results.

## 4. Experiments

### 4.1. Datasets

Three public datasets, namely CoNSep [5], Lizard [28], and PanNuke [29,30], are used to validate the performance of AER-Net. CoNSep and Lizard are colorectal cancer datasets, while PanNuke is a pan-cancer dataset containing a variety of different cancer types.

The CoNSep dataset is a colorectal nuclear segmentation and phenotypes dataset, consisting of 41 H&E stained images scanned at 40× magnification. Each image in the dataset has a size of 1000×1000 pixels. Referring to the setup of the paper [5], the nuclear classes in the CoNSep dataset are divided into four classes which are epithelial, inflammatory, spindle-shaped, and miscellaneous. The training set consists of 27 images, while the test set comprises 14 images.

The Lizard dataset is the largest known dataset for nuclear segmentation and classification, comprising histology image regions of colon tissue from six different data sources. The dataset images are at 20× magnification and provide nuclear classification labels. The dataset is labeled with six different types of nuclei including neutrophil, epithelial, lymphocyte, plasma, eosinophil, and connective. For the Lizard dataset, we use the data provided in CoNIC 2022 [6], which contains 4981 images with a size of 256×256 pixels. In order to better evaluate AER-Net, we divided 4981 images into 2988 as a training set, 996 as a validation set, and 997 as a test set by stratified random sampling.

The PanNuke dataset contains H&E staining images of 19 different tissue types with the nuclear classes divided into five classes. The dataset is organized into three folds, comprising 2656, 2523, and 2722 images, respectively. Each image has a size of 256×256 pixels. We use fold1 as the training set, fold2 as the validation set, and fold3 as the test set.

### 4.2. Evaluation Metrics

#### 4.2.1. Nuclear Instance Segmentation Evaluation

The evaluation metric for the instance segmentation uses the Panoptic Quality (PQ), which was originally proposed by Kirillov et al. [31].

(15) $$\begin{array}{c} PQ=\underset{\begin{array}{c} \mathrm{Detection}\;\mathrm{Quality}(\mathrm{DQ}) \end{array}}{\underset{︸}{\frac{\left|TP\right|}{\left|TP\right|+\frac{1}{2}\left|FP\right|+\frac{1}{2}\left|FN\right|}}}\times \underset{\begin{array}{c} \mathrm{Segmentation}\;\mathrm{Quality}(\mathrm{SQ})\; \end{array}}{\underset{︸}{\frac{\sum_{(x,y)\in TP}IoU\left(x,y\right)}{\left|TP\right|}}} \end{array}$$

where *x* is the ground truth, *y* is the predicted outcome, and IoU is the intersection over the union. If IoU(x,y)>0.5, it is assumed that *x* and *y* are a unique matching pair. TP denotes matched pairs, FN denotes unmatched Ground Truth (GT) segments, and FP denotes unmatched prediction segments. Panoptic Quality (PQ) can be visualized as the product of Detection Quality (DQ) and Segmentation Quality (SQ).

#### 4.2.2. Nuclear Classification Evaluation

We use the detection metric $F_{d}$ and the classification metric $F_{t}$ for the evaluation of the nuclei classification task. $F_{d}$ is defined as follows:

(16) $$\begin{array}{c} F_{d}=\frac{2TP_{d}}{2TP_{d}+FP_{d}+FN_{d}} \end{array}$$

where $TP_{d}$ denotes correctly detected instances, $FN_{d}$ denotes incorrectly detected GT instances, and $FP_{d}$ denotes over-detected instances. $TP_{d}$ is further divided into correctly classified type t instances $TP_{t}$, correctly classified non-type t instances ($TN_{t}$), incorrectly classified type t instances ($FP_{t}$), and incorrectly classified non-type t instances ($FN_{t}$), which are used to compute $F_{t}$ for the classification task.

(17) $$\begin{array}{c} F_{t}=\frac{2(TP_{t}+TN_{t})}{2\left(TP_{t}+TN_{t}\right)+2FP_{t}+2FN_{t}+FP_{d}+FN_{d}} \end{array}$$

### 4.3. Implementation Details

We implemented our framework using PyTorch on a workstation that has an NVIDIA GeForce RTX 3090 GPU (24G). In the experiments with the CoNSep dataset, the input size of our model and HoverNet is 270×270 pixels, and the output size is 80×80 pixels. For the experiments on the Lizard dataset, the input and output sizes of both AER-Net and HoverNet are 256×256 pixels. In the experiments on the PanNuke dataset, the input size of AER-Net and HoverNet is 256×256 pixels, and the output size is 164×164 pixels. During model training, various data augmentation methods such as flipping, Gaussian blurring, and median blurring are employed. The whole training process is divided into two stages each with 50 epochs, and the same settings are applied in each stage. In the first stage, we do not freeze the encoder but train it directly. The batch size is four for both the first and second stages of the training process. The model parameters are initialized with pre-trained weights from the ImageNet [32] dataset, we use the Adam optimization method, and the initial learning rate is 0.0001, which will be reduced to 0.00001 after 25 epochs. For the loss function parameters in the training of the CoNSep dataset, we set $\beta_{2}$ to 2 and used default values for all other parameters. For Lizard and PanNuke dataset training, the loss function parameters all use default values. Note that for the Pannuke dataset, ARRM utilizes only two pooling operations, which is because the output of the model for the Pannuke dataset is 164×164 pixels.

### 4.4. Experimental Results

We compare the AER-Net with other state-of-the-art methods on the CoNSep dataset, as illustrated in Table 1. The experimental results for the Lizard dataset are shown in Table 2. In addition, we compared the baseline (HoverNet) with the AER-Net on the PanNuke dataset to validate the predictive ability of our model on different tissues.

**Figure 6:**
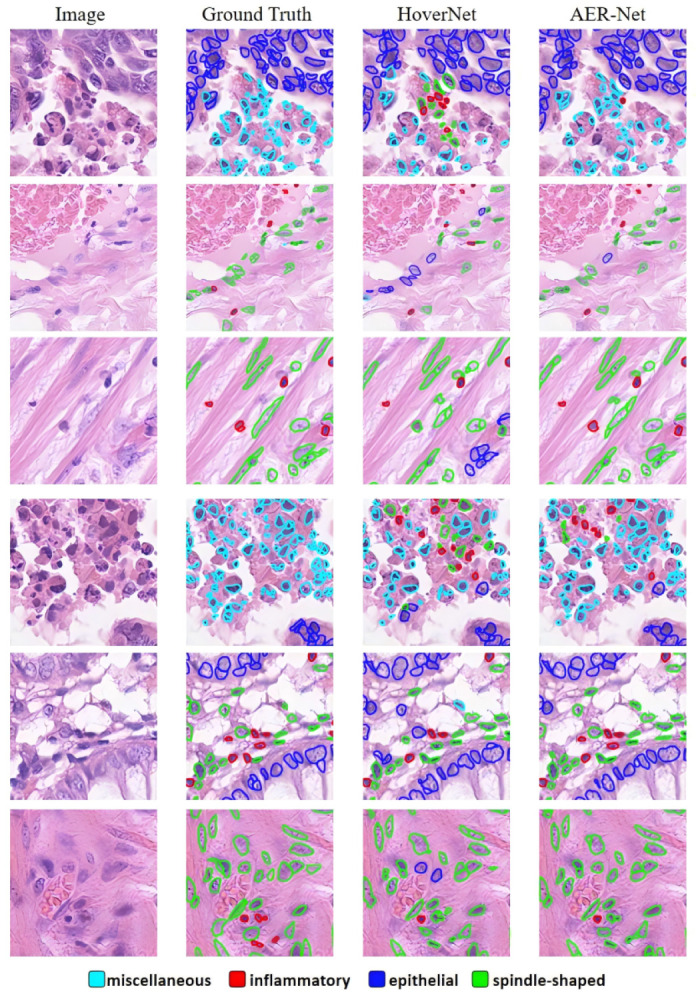
Example visualization results on the CoNSeP dataset.

Firstly, experiments were conducted on the CoNSep dataset, and the results in Table 1 reveal that AER-Net achieved the best performance on the CoNSep dataset. AER-Net achieved the highest PQ of 0.556, which is 3.5% higher than the second highest value. This result indicates that AER-Net achieves excellent segmentation performance on smaller datasets such as CoNSep. In the CoNSep dataset, there are four types of nuclei: miscellaneous, inflammatory, epithelial, and spindle-shaped nuclei, corresponding to $F_{m}$, $F_{i}$, $F_{e}$, and $F_{s}$ in Table 1. The number of nuclei in the miscellaneous class is lower, and hence the $F_{m}$ is lower for many methods. Notably, AER-Net still performs well in classifying miscellaneous nuclei, achieving an $F_{m}$16.2% higher than the result of HoverNet. This indicates that our proposed method can effectively address the class imbalance of nuclei instances in the task of nuclei segmentation and classification. The comparison between AER-Net and HoverNet results is depicted in Figure 6, where it can be observed that the segmentation results and the predictions of nuclear classification are more accurate in AER-Net compared with HoverNet.

**Figure 7:**
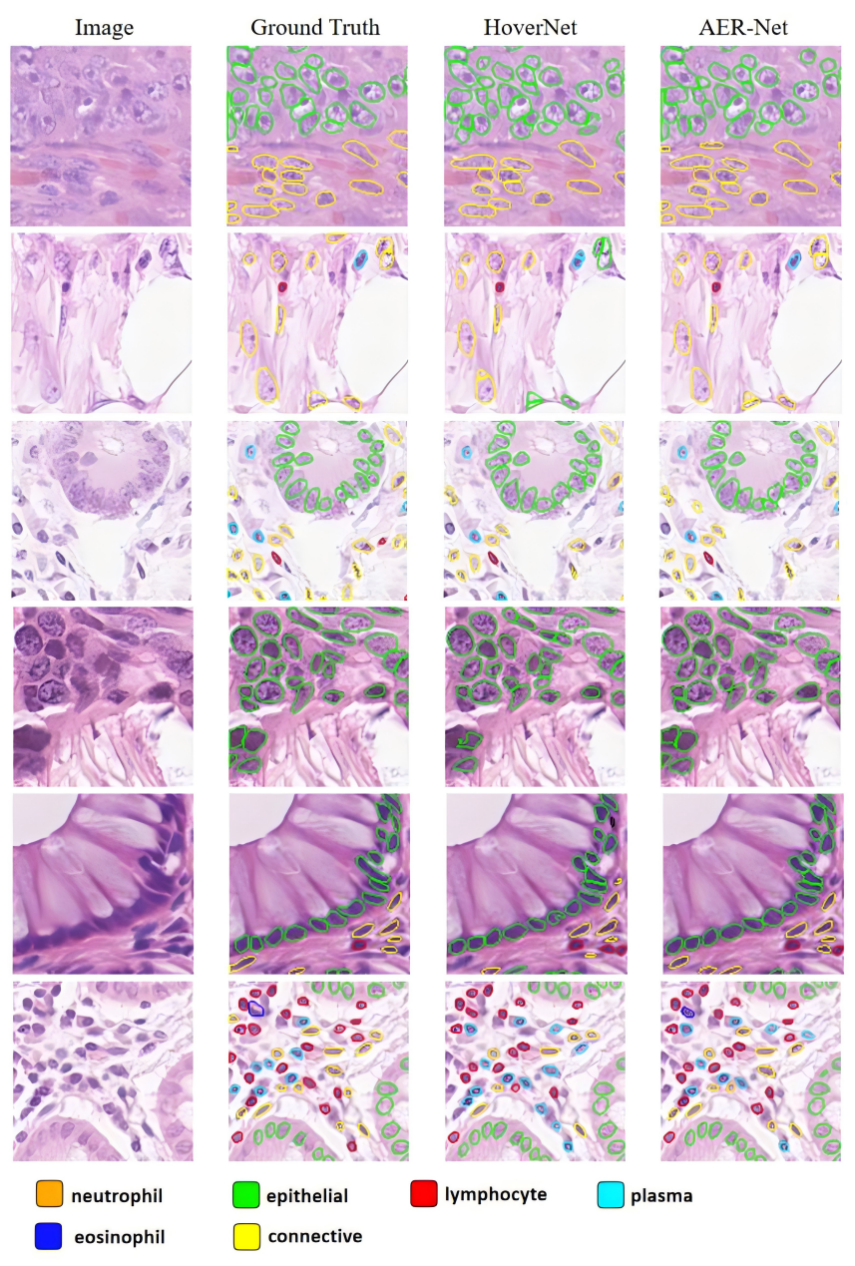
Example visualization results on the Lizard dataset.

We conducted experiments on a larger dataset, Lizard, and the experimental results are presented in Table 2. $F_{ne}$, $F_{ep}$, $F_{l}$, $F_{p}$, $F_{eo}$, and $F_{c}$ denote the $F_{1}$ classification scores for neutrophil, epithelial, lymphocyte, plasma, eosinophil, and connective nuclei, respectively. The experimental results show that AER-Net achieves a PQ of 0.608. It can be observed that AER-Net demonstrates the best performance in terms of $F_{d}$, $F_{ne}$, $F_{ep}$, $F_{l}$, $F_{p}$, $F_{eo}$, and $F_{c}$. In the Lizard dataset, the number of nuclei in the eosinophil and neutrophil classes is significantly smaller compared with the nuclei in the other classes. It is noteworthy that AER-Net demonstrated significant improvement in the eosinophil and neutrophil classes with a 3.1% improvement in $F_{eo}$ and a 6.0% improvement in $F_{ne}$. This further validates the potential of AER-Net for nuclear classification. Figure 7 illustrates the results of AER-Net compared with HoverNet. It can be observed that despite the numerous challenges in the image, AER-Net still achieves excellent results.

A comparison of Table 1 and Table 2 reveals that with the increase in the dataset, AER-Net can also achieve better results on the nuclei segmentation and classification tasks. In the small-scale dataset CoNSep, the number of samples is limited, and AER-Net can utilize the advantages of the residual refinement module to significantly improve the results of nuclei segmentation and classification. In large-scale datasets, where the diversity and complexity of data increase, AER-Net still performs well. Although the improvement of segmentation performance is not obvious in the Lizard large-scale dataset, the classification performance is still significantly improved, which shows that AER-Net can adapt to data of different scales and complexity with stronger generalization ability.

In order to further validate the segmentation and classification ability of AER-Net for nuclei of different tissues, we performed experiments on the PanNuke dataset, as shown in Table 3. $F_{n}$, $F_{i}$, $F_{c}$, $F_{dead}$ and $F_{e}$ denote the $F_{1}$ classification scores for neoplastic, inflammatory, connective tissue, dead and non-neoplastic nuclei, respectively. It can be seen that AER-Net achieved the highest PQ of 0.644, indicating that AER-Net is also competitive in nuclear instance segmentation across different tissues. Moreover, as depicted in Table 3, it can be observed that AER-Net demonstrates improved performance in nuclear classification compared with HoverNet. In conclusion, AER-Net still achieves excellent performance in nuclear segmentation and classification in different tissues. The F1 scores take into account the type of nuclei, so while the improvements in the PQ and Fd metrics are not significant, the improvement in the F1 scores for each class also demonstrates the enhancement of the AER-Net in the overall task of nuclear segmentation and classification.

### 4.5. Ablation Study

To analyze the performance of each module, we further conducted ablation experiments on the CoNSep dataset. We used the same experimental settings for each method in the ablation experiments to ensure a fair comparison. Baseline denotes HoverNet, AEncoder denotes an encoder that utilizes the CSAR module based on the Baseline, and ARRM indicates the use of the ARRM module based on the baseline. AEncoder&ARRM is our model ARE-Net including CSAR and ARRM. The results of our experiments are presented in Table 4. A powerful encoder is essential for nuclear segmentation and classification, and the experimental results show that the channel and spatial attention-enhanced encoder we used significantly improves the predictive performance of the model. The best performance of the model is observed when both the attention-enhanced encoder and the attention-enhanced residual refinement module are used.

We also performed ablation experiments on the weights of the auxiliary loss in the loss function, where L1 denotes the auxiliary loss. The experimental results are shown in Table 5, and it is observed that the best performance is achieved when α is set to 1. It should be pointed out that the weight of auxiliary loss is only a hyperparameter, which should be adjusted accordingly for different datasets.

In addition, we tried to adjust the weights of the loss function for each branch in Equation (2). The experimental results are shown in Table 6. It can be seen that the results are better when the weights of $L_{a}$, $L_{b}$, $L_{c}$ are all 1 that is our used in the comparative experiments and independent validation.

### 4.6. Independent Validation

The differences in the datasets make it difficult to apply a model developed on one dataset to another dataset to assess its generalizability. DigestPath and CRAG are two subsets of the Lizard dataset. CRAG contains image regions extracted from the whole slide image (WSI) of the University Hospital Coventry and Warwick (UHCW). DigestPath contains image regions of biopsy samples from four different hospitals in China. Inspired by the work of Xiao et al. [21], we first divide CRAG into training and validation sets for training and use DigestPath as a test set. Then, we divide DigestPath into training and validation sets for training and use CRAG as a test set. The experimental results are presented in Table 7 and Table 8. It can be observed that our method achieved the best overall results in both sets of experiments. Furthermore, it is worth noting that the improvement of our method is more pronounced when experimenting on datasets with less data.

## 5. Conclusions

The accurate segmentation and classification of nuclei in histology images are crucial for providing valuable insights for the diagnosis and prognostic analysis of colorectal cancer. Nevertheless, histology images often contain numerous clusters of nuclei, posing challenges in accurately predicting their boundaries. Moreover, the class imbalance among nuclei presents another significant challenge in the field. In this study, we introduce AER-Net, which is a method designed to achieve the precise segmentation and classification of nuclei. Firstly, we utilize an attention-enhanced encoder module to enhance the feature extraction within the encoder. Secondly, we incorporate ARRM modules into each decoder branch to enhance the precision of our predictions, refining the initial coarse results for greater accuracy. Additionally, we address the challenge of classes imbalance among nuclei in pathology images by implementing a loss function that combines cross-entropy loss and generalized dice loss. Experimental results show that AER-Net performs well in nuclear segmentation and classification with excellent generalization ability. In the future, the model performance can be further improved by combining the AER-Net method with unsupervised methods to utilize more pathology image data.

## Figures and Tables

**Table 1:** Comparative experimental results on the CoNSep dataset.

Model	$F_{d}$	$F_{e}$	$F_{i}$	$F_{s}$	$F_{m}$	PQ
SC-CNN [19]	0.608	0.306	0.193	0.175	0.000	-
DIST [14]	0.712	0.617	0.534	0.505	0.000	0.372
Micro-Net [11]	0.743	0.615	0.592	0.532	0.117	0.430
Mask-RCNN [15]	0.692	0.595	0.590	0.520	0.098	0.450
HoverNet [5]	0.752	0.633	0.531	0.545	0.358	0.521
AER-Net	**0.782**	**0.682**	**0.626**	**0.597**	**0.520**	**0.556**

**Table 2:** Comparative experimental results on the Lizard dataset.

Model	$F_{d}$	$F_{\mathrm{ne}}$	$F_{\mathrm{ep}}$	$F_{l}$	$F_{p}$	$F_{\mathrm{eo}}$	$F_{c}$	PQ
Unet [10]	0.780	0.141	0.673	0.611	0.299	0.280	0.620	0.456
Mask-RCNN [15]	0.766	0.229	0.751	0.601	0.365	0.316	0.587	0.531
HoverNet [5]	0.821	0.220	0.760	0.638	0.396	0.349	0.620	0.607
AER-Net	**0.825**	**0.280**	**0.785**	**0.649**	**0.421**	**0.380**	**0.654**	**0.608**

**Table 3:** Comparative experimental results on the PanNuke dataset.

Model	$F_{d}$	$F_{n}$	$F_{i}$	$F_{c}$	$F_{\mathrm{dead}}$	$F_{e}$	PQ
HoverNet [5]	0.791	0.561	0.472	**0.405**	0.160	0.406	0.643
AER-Net	**0.791**	**0.593**	**0.493**	0.402	**0.201**	**0.500**	**0.644**

**Table 4:** The results of different modules on the CoNSep dataset.

Model	$F_{d}$	$F_{e}$	$F_{i}$	$F_{s}$	$F_{m}$	PQ
Baseline	0.752	0.633	0.531	0.545	0.358	0.521
AEncoder	0.774	0.656	**0.628**	0.595	0.443	0.551
ARRM	0.763	0.637	0.575	0.569	0.382	0.546
AEncoder&ARRM	**0.782**	**0.682**	0.626	**0.597**	**0.520**	**0.556**

**Table 5:** The results of experiments with different α on the CoNSep dataset.

Model	$F_{d}$	$F_{e}$	$F_{i}$	$F_{s}$	$F_{m}$	PQ
$L_{2}$	0.769	0.658	**0.634**	0.581	0.488	0.550
0.2$L_{1}$ + $L_{2}$	0.772	0.671	0.615	0.582	0.426	0.549
0.4$L_{1}$ + $L_{2}$	0.760	0.649	0.614	0.583	0.488	0.548
0.6$L_{1}$ + $L_{2}$	0.773	0.659	0.614	0.587	0.516	0.551
0.8$L_{1}$ + $L_{2}$	0.770	0.672	0.609	0.587	0.481	0.554
$L_{1}$ + $L_{2}$	**0.782**	**0.682**	0.626	**0.597**	**0.520**	**0.556**

**Table 6:** The results of experiments with different weights for each branch in loss function Equation (2).

Model	$F_{d}$	$F_{e}$	$F_{i}$	$F_{s}$	$F_{m}$	PQ
2$L_{a}$ + $L_{b}$ + $L_{c}$	0.769	0.658	0.622	0.573	0.504	0.548
$L_{a}$ + 2$L_{b}$ + $L_{c}$	0.750	0.615	0.574	0.559	0.391	0.543
$L_{a}$ + $L_{b}$ + 2$L_{c}$	0.770	0.667	0.622	0.596	0.505	0.545
2$L_{a}$ + 2$L_{b}$ + $L_{c}$	0.774	0.663	0.589	0.585	0.441	0.553
2$L_{a}$ + $L_{b}$ + 2$L_{c}$	0.765	0.657	**0.631**	0.590	0.482	0.551
$L_{a}$ + 2$L_{b}$ + 2$L_{c}$	0.755	0.652	0.613	0.578	0.476	0.546
$L_{a}$ + $L_{b}$ + $L_{c}$	**0.782**	**0.682**	0.626	**0.597**	**0.520**	**0.556**

**Table 7:** The results of the independent validation experiments utilized CRAG as the training set and DigestPath as the test set.

Model	$F_{d}$	$F_{\mathrm{ne}}$	$F_{\mathrm{ep}}$	$F_{l}$	$F_{p}$	$F_{\mathrm{eo}}$	$F_{c}$	PQ
HoverNet [5]	0.740	**0.021**	0.560	0.417	0.202	0.149	0.391	0.528
AER-Net	**0.770**	0.013	**0.620**	**0.521**	**0.231**	**0.163**	**0.460**	**0.554**

**Table 8:** The results of the independent validation experiments utilized DigestPath as the training set and CRAG as the test set.

Model	$F_{d}$	$F_{\mathrm{ne}}$	$F_{\mathrm{ep}}$	$F_{l}$	$F_{p}$	$F_{\mathrm{eo}}$	$F_{c}$	PQ
HoverNet [5]	0.725	0.045	0.588	0.439	0.225	0.070	0.357	0.405
AER-Net	**0.729**	**0.072**	**0.627**	**0.462**	**0.265**	**0.104**	**0.410**	**0.415**

## Data Availability

Data are contained within the article.
